# Attachment histories and futures: reply to Vicedo’s ‘Putting attachment in its place’

**DOI:** 10.1080/17405629.2018.1502916

**Published:** 2018-08-02

**Authors:** Robbie Duschinsky, Marinus Van Ijzendoorn, Sarah Foster, Sophie Reijman, Francesca Lionetti

**Affiliations:** a The Primary Care Unit, Institute of Public Health, University of Cambridge School of Clinical, Cambridge, UK; b Graduate School Social and Behavioural Sciences, Leiden University, The Netherlands; c Department of Psychology, Education, and Child Studies, Erasmus University Rotterdam, Rotterdam, The Netherlands; d Social Work, Education & Community Wellbeing, Northumbria University, Newcastle, UK; e School of Biological and Chemical Sciences Queen Mary, University of London, London, UK

## Abstract

For Vicedo, ‘putting attachment in its place’ seems to entail two aspects. The first is working to understand the rise of attachment theory and its place within the history of knowledge practices. The second is to criticize the validity of attachment theory. In this reply, we appraise three criticisms made by Vicedo of attachment theory, chosen as points for sustaining a dialogue. Our main point in this reply is that, in excluding the work of attachment researchers after Ainsworth from consideration, Vicedo’s work is not yet able to properly ‘put attachment in its place’, in either sense of the phrase. At most, she puts Bowlby in the 1950s–1960s in his place, but without speaking effectively to subsequent attachment research. In our view, not just the validity, but the very meaning of attachment as a scientific research programme cannot be understood outside of its temporal context, and the relationship this entails between theory and research, past and future.

## 1 History and attachment research

In *The Nature and Nurture of Love*, Vicedo states that ‘the scientific evidence in support of attachment theory has been insuffcient and is deeply flawed’ (2013, p. 238), a position that Vicedo continues to argue in ‘Putting attachment in its place’. We agree that it is an appropriate part of the role of a historian of science to appraise the evidence under discussion. However, issues as well as insights may arise when a historian treats evidence of the contingent, social and messy origins of ideas as evidence of their lack of scientific validity. This is to miss the distinction, always relative but nonetheless often meaningful, between the generation of ideas and the slow nature of work to appraise their validity (cf. Schickore & Steinle, 2006). We fully acknowledge that most of Bowlby’s ideas were not well-grounded in adequate supporting evidence, were influenced by contemporary ideologies, and that caution is needed in using those that have not seen adequate testing. This is often the case with the development of early theories across the sciences (Collins, 2004).

Furthermore, some of Bowlby’s hypotheses have been very valuable, producing knowledge that is reliable and a good basis for the design of policies such as family rooming-in during a child’s hospitalization. He was also right to contest the overemphasis on fantasy at the expense of the actuality of child experiences in the psychoanalytic thought of his day. He was absolutely right that child institutionalization is generally harmful and emotionally damaging (Dozier et al., 2014; Lionetti, Pastore, & Barone, 2015) although convincing empirical evidence was lacking 70 years ago (Bowlby, 1953). However, Bowlby was also wrong on many accounts, and Vicedo appropriately points to occasions where he expressed undue and unwarranted confidence in certain claims with potentially damaging social effects. One area where later research has rectified early theory has been in relation to daycare. Bowlby underestimated the role of child factors in the experience of daycare; researchers have found that infants with a prior tendency to be more readily upset appear to be more affected by the quality of care they experience – both negatively *and* positively – than other children (Pluess & Belsky, 2009). Bowlby’s thinking also radically underspecified the concepts of ‘separation’ and ‘deprivation’. As a consequence he was wrong that daycare is an experience of a similar kind as institutionalization, although his emphasis on continuity and sensitivity of care turned out empirically to be important factors for quality daycare. Longitudinal research following 1,153 children from infancy to adolescence found that quality day-care for young children whose mothers are highly stressed confers a net benefit (NICHD, 1997).

## 2 Continuities or discontinuities across generations of researchers

The tendency in Vicedo’s writings to assume uncomplicated continuity from attachment research from the mid-1950s to the present has been criticized by both historians and psychologists. Midgley (2014, p. 266) describes Vicedo’s account of attachment theory as ‘polemical’ since it seems to wish to debunk attachment research, but displays ‘lack of attention to the work of Mary Main and others in the last forty years’, and is therefore tellingly ‘outdated’, without purchase on the present (268). In her paper on ‘Putting attachment in its place’, as well as in her other work that we have seen, Vicedo excludes mention of subsequent developments in attachment theory by Ainsworth’s students, or their students. Vicedo makes no mention of the cross-cultural work that attachment researchers have conducted. And she makes no citation of the field’s journal *Attachment & Human Development* or the field’s compendium, the *Handbook of Attachment* (now in its third edition; e.g. Mesman, van IJzendoorn, & Sagi-Schwartz, 2016). Other commentators have similarly observed that Vicedo’s criticisms do not strike home because she conflates Bowlby with attachment theory and research, and underplays developments in Bowlby’s thinking across time. Vicedo does not acknowledge that ‘one can jettison the idea of mother love as instinctual, as well as the fixation on the mother as the crucial attachment figure, without discarding all of the theory’s insights’ (Plant, 2015, p. 459). The idea of mother love as instinctual was an early formulation especially to be found in Bowlby’s *Child Care and the Growth of Love* (1953). Yet at least from 1964 (see PP/Bow/H.147), whilst there may be a short period where one or another caregiver is preferred when an infant is distressed, for Bowlby the attachment system was conceptualized as organized by the expectation that distributed caregiving may be available, and attachments made to various caregivers. Bowlby’s last published work explicitly states the attachment system ‘contributes to the individual’s survival by keeping him or her in touch with one or more caregivers.’ (1991, p. 306). Bowlby (1969, p. 303) wrote ‘it has sometimes been alleged that I have expressed the view . . . that mothering “cannot be safely distributed among several figures” (Mead, 1962). No such views have been expressed by me.’ Vicedo’s inclusion of Mead’s allegation in ‘Putting attachment in its place’, but not Bowlby’s direct reply to it, suggests partiality.

Later generations of attachment researchers have not held mother love to be instinctual, nor have they regarded it as necessary that the mother would be a child’s sole attachment figure. The work of Sarah Hrdy (e.g. 2009) is an important contemporary influence in this regard. It is true, as Vicedo states, that siblings as caregivers have not yet received adequate attention in attachment research (but see the recent paper on infant–mother and infant–sibling attachment in Zambia by Mooya, Sichimba, & Bakermans-Kranenburg, 2016). For decades now, Bowlby’s ideas have been elaborated in generating testable hypotheses on the development of attachment in social networks (e.g. Goossens & Ijzendoorn, 1990). In ‘Putting attachment in its place’ Vicedo states that the bulk of attachment research ‘has focused on testing a single variable (security), and has appealed to a single explanatory factor (maternal sensitivity)’ (692). However this has not been true for many decades as various meta-analyses document (e.g., Cyr, Euser, Bakermans-Kranenburg, & van Ijzendoorn, 2010). Nor was it inappropriate in the 1980s for this core hypothesis to have been a focus for a time, since extensive replication was needed in order to establish and, indeed, substantially qualify the standing of Ainsworth’s empirical claims (Verhage et al., 2016).

## 3 The caregiving system

Another criticism of attachment research made by Vicedo which we feel has a certain purchase, but also limitations, are her claims relating to the conceptualization of caregiving. In *The Nature and Nurture of Love*, Vicedo states that ‘In arguing that the mother is designed to fulfill her child’s instinctual needs Bowlby transformed maternal love and care from a personal choice entailing devotion, work, patience, dedication, and not a few renunciations into a natural product of a woman’s biological constitution’ (2013, p. 90). In our assessment, this criticism holds well for Bowlby’s popular writings of the 1950s. It does not hold quite so well for Bowlby’s later thought, and it is a poor characterization of attachment research over the past decades. The adult caregiving behavioral system has been theorised as a construct conceptually and evolutionary distinct from the child attachment system, and as highly dependent on social support and cultural processes for whether, when and how they are deployed. And empirical operationalisations of the concept of caregiver sensitivity explicitly make space for situation-specific and child-related factors rather than prescribing concrete behaviors.

In ‘Putting attachment in its place’, Vicedo argues that ‘contrary to what attachment theory considers normative, parents in many communities try to avoid attaching to their infants right away.’ (2017, p. 693) Parental avoidance of emotional connection with their infant is certainly contrary to the normative picture of *Child Care and the Growth of Love*, and in the 1950s Bowlby sometimes, confusingly, used the term ‘attachment’ to refer to what he would later distinguish as the caregiving system. However, Vicedo’s observation is in line with, not contrary to, attachment theory over the past decades. The idea that parents may use strategies to modulate the expression of caregiving behaviour, such as directing attention away from their child if they suspect that the child could be a source of distress, is well in line with the ideas of Mary Main and others at least from 1992. As well as theory, there has been empirical research exploring predictable individual differences regarding how children respond to cultural differences in caregiving sensitivity (Mesman et al., 2016). One relevant study that Vicedo may be interested to consider is Mesman et al. (2016b), who found significant differences in beliefs about the appropriateness of sensitive caregiving between cultural groups. Some of this effect was accounted for by group variations in poverty.

## 4 The validity of the ainsworth strange situation

A third set of criticisms made of attachment theory by Vicedo concerns the validity of the Strange Situation procedure. Vicedo argues that reliance on the Strange Situation has ‘led developmental psychologists to a distorted vision of children because it ignored the role of context in child development.’ (2017, p. 689). Again, Vicedo’s claims have some relevance but are substantially outdated. It is true that too few attachment researchers include detailed ethnographic observations of naturalistic contexts when conducting their research, because there might be more to discover and grounded hypotheses to be developed. Nonetheless, there has been substantial study of children’s social contexts and how these affect the development of their attachments (e.g., Cyr et al., 2010; Sroufe et al. 2005). Furthermore, infant behaviour in the Strange Situation has repeatedly been found to be associated with extensive observations of dyadic interactions in naturalistic settings (Pederson, Gleason, Moran, & Bento, 1998; True, Pisani, & Oumar, 2001). Alternative attachment measures such as the Attachment Q-Sort have been widely used since the 1980s in homes rather than in the strange situation of the laboratory (Waters & Deane, 1985), but they do not receive mention by Vicedo.

Another issue of validity raised by Vicedo, and the particular focus of ‘Putting attachment in its place’, is the cross-cultural applicability of attachment theory and methods. The article continues the critique outlined in *The Nature and Nurture of Love*, that ‘Whether in a poverty-stricken family in an African village, in a middle-class suburban American home, or in the very strange situation of an infant left alone with a stranger in a psychological laboratory, Ainsworth discerned the same “patterns of attachment behaviour”.’ (2013, p. 207). However, Vicedo seems unaware that the phrase ‘patterns of attachment’ changed meaning between the Uganda ethnography and the Baltimore study. In *Infancy in Uganda* (1967, p. 332), Ainsworth writes of ‘differential crying’ and ‘lifting arms in greeting’ as examples of patterns of ‘attachment behaviour’. No claim was made that all children around the world show these behaviours when distressed. Indeed, Ainsworth’s interest was in the fact that not even all children in the same family show these behaviours towards their caregiver, and that changes in family environment altered the likelihood of these behaviours being displayed. There is an important distinction between discrete behaviours and the organization of behaviours (Sroufe & Waters, 1977). The phrase ‘patterns of attachment’ was later repurposed by Ainsworth to refer to her classifications for the Strange Situation, but none of these are based necessarily on discrete behaviours. An infant can be classified as secure, for example, without ever approaching the caregiver or getting in contact; an infant can be classified as resistant without a display of anger. Indeed, these classifications were labeled A, B, and C by Ainsworth to avoid premature normative connotations with ‘secure’ or ‘insecure’ attachments. And later work by various researchers including Hinde, Main and Belsky emphasized the adaptive role of ‘insecure’ attachments in less than optimal rearing niches.

We know of no passage where Ainsworth stated an expectation that all infants would fit the three classifications found in her Baltimore study, and in a letter to Bowlby of the 10 March 1984 (Bowlby Archive PP/BOW/B.3/8) she stated explicitly that she was ‘uneasy’ at the very thought, and that further cross-cultural research would be needed before she would even take the proposal seriously. She was enthusiastic about cross-cultural research, for instance praising the work of her student Bob Marvin and colleagues for their study with the polymatric Hausa of Nigeria. In an interview, Ainsworth stated ‘I think that environmental influences play no significant role in the infant’s basic need for an attachment figure who can be trusted. But culture-related differences in ecologies and expectations will certainly affect how some specific aspects of that organization are expressed’ (Ainsworth & Marvin, 1995, p. 8). Vicedo ‘puts attachment in its place’ in claiming that attachment researchers have failed to engage with cross-cultural research, but this claim is only possible because her analysis stops before the 1990s. She is right that there is need for further crosscultural research, and attachment researchers have acknowledged this (e.g. Mesman et al., 2016). Yet decades of cross-cultural research have demonstrated the applicability of the Ainsworth classifications, though not their exhaustiveness. In ‘Putting attachment in its place’, Vicedo warmly cites claims that the Strange Situation ‘cannot be used to study children in nonWestern cultures’ (2017, p. 690) but she ignores the large number of studies, reviewed in the *Handbook of Attachment*, conducted with the Strange Situation in a variety of non-WEIRD countries and cultures – including societies characterized by high levels of alloparenting. Crosscultural attachment research does suggest that there are some general aspects to attachment, but this does not preclude very significant culture specific responses (Mesman et al., 2016). The relative cross-cultural validity of a research instrument and the cultural specificity of the things it seeks to measure does not represent a contradiction in terms, as Vicedo implies.
